# Amazonian cosmologies, plant–human relations, and the colonial entanglements of Indigenous artifacts

**DOI:** 10.1177/00732753261423335

**Published:** 2026-04-04

**Authors:** Raphael Uchôa

**Affiliations:** Museu de Astronomia e Ciências Afins, Brazil; Center for Social Studies, University of Coimbra, Portugal

**Keywords:** Colonial science, Amazonian artifacts, Indigenous epistemologies, museums, global histories, plant–human relations

## Abstract

This article examines the deep entanglement of Amazonian artifacts housed in European museums, colonial science, and Indigenous cosmologies. By situating specific collection- and curation processes within their historical contexts, it reveals how scientific practices were intricately tied to colonial expansion, functioning as tools to legitimize and sustain European hegemony. Analysis of Amerindian masks and botanical specimens from the Amazon Basin – two distinct yet interrelated sets of Amazonian artifacts currently held in European institutions – highlights how colonial extractive practices, often reliant on Indigenous slave labor, and geopolitical dynamics shaped the acquisition, interpretation, and display of Indigenous materials. The article also critiques how European scholars and political regimes have treated regions such as the Amazon and Africa as interchangeable, enforcing a reductive, hierarchical view of non-European cultures and reducing the diversity of Indigenous societies to objects of control and categorization. By interweaving insights and critiques by Indigenous and Amazonian intellectuals, particularly Yanomami shaman Davi Kopenawa, and drawing on cosmologies of the Rio Negro region, in the Amazon, this study calls for a fundamental reassessment of knowledge-production frameworks shaped by colonialism and scientific exploration. It underscores the inadequacies of Eurocentric approaches and advocates for the transformative potential of Indigenous epistemologies – not as peripheral perspectives, but as foundational contributions that challenge, reshape, and enrich global histories of science.

Another time, I was taken to visit a vast house to which the white people gave the name of museum.^1^

Davi Kopenawa, a Yanomami shaman

## Introduction

In 2022, I visited the Science Museum at the University of Coimbra, one of Europe’s oldest universities. As a historian of science, and as someone born and raised in the Brazilian Amazon, the experience offered a sobering illustration of how colonial legacies continue to shape museum practices and, ultimately, science communication. Among the objects on display in an exhibition titled “Cabinet of Curiosities” an Amazonian zoomorphic mask from the Jurupixuna people stood alongside instruments of early European science. There was no contextual information, no reference to the communities from which these objects were taken, nor any indication of the political, historical, or cosmological dimensions that shaped their original meaning. The juxtaposition of Amerindian artifacts, African relics, animal specimens, and phrenological casts evoked a vision of imperial classification at its most uncritical – and most violent – expression. Despite growing calls for decolonial curatorship across Latin America, exhibitions like this persist – remnants of a museography and a scientific worldview that continue to frame Indigenous material culture as exotic, interchangeable, and mute.^2^

This experience crystallized the broader questions I explore in this article. The Amazon region presents a critical site for investigating non-Western knowledge of nature and the ongoing epistemic consequences of colonial scientific practices. While anthropologists have extensively examined contemporary Amerindian ontologies in Latin America, historical analyses have only unevenly centered Indigenous conceptualizations of nature and their entanglement with colonial science, particularly in the Amazonian context.^3^ This gap is partly due to the methodological challenges posed by oral traditions, which are often accessible only through objects and records filtered by European naturalists, missionaries, and collectors.^4^ Such materials are deeply inscribed with Euronormative hierarchies and reflect foundational asymmetries in the framing of concepts like “truth,” “nature,” and “science.”

Even in the wake of the so-called material turn, much of the historiography of science continues to privilege text-based sources, often sidelining oral, embodied, and cosmological dimensions of Indigenous knowledge systems.^5^ In the Amazon, where material culture constitutes one of the few remaining traces of historical epistemologies, objects collected during colonial expeditions between the late eighteenth and early twentieth centuries offer a valuable – albeit contested – entry point.^6^ But can these artifacts, dislocated from their original contexts, be meaningfully interpreted through the lens of the history of science? Or are they better suited to anthropological or museological inquiry? More critically, how can Indigenous perspectives – often rooted in myth, kinship, and ritual – inform these analyses without being reduced to symbolic footnotes or cultural folklore?^7^

This analysis calls for a dual focus: not only on the epistemological status of these artifacts, but also on the historical conditions that enabled their collection, transit, and displacement – including the often-overlooked role of Indigenous labor in the making of scientific archives. The transfer of Amazonian materials to European institutions was shaped by complex dynamics of asymmetrical collaboration, coercion, and epistemic translation. These processes demand renewed methodological scrutiny within the history of science, particularly in light of the ways they have produced enduring regimes of classification and display. The Coimbra Science Museum and Kew Gardens are not presented here as representative of all European institutions. Rather, they serve as analytically revealing sites where tensions between imperial taxonomies and Indigenous cosmologies become especially visible, illuminating the epistemic frictions at the heart of these collections. Rather than treating Indigenous knowledge systems as ethnographic objects to be contextualized, this article engages Indigenous intellectual production as a source of conceptual provocation for the history of science itself. In doing so, it explores how Indigenous critiques of museums, objects, and archives unsettle established evidentiary regimes and invite methodological recalibration.

This article suggests that examining these dynamics through the analytical lens of the history of science – attuned to the transit, classification, and epistemic translation of objects – offers a distinctive perspective on how imperial knowledge regimes did not merely ignore, but actively transformed, suppressed, and appropriated Indigenous cosmologies.^8^ This begs a crucial disciplinary question: what methodological and conceptual tools can historians of science mobilize to apprehend the material, symbolic, and cosmological dimensions of these objects beyond their archival or taxonomic traces? And how they might – and why they should – recalibrate their frameworks in response to the epistemic provocations articulated by Indigenous artists, activists, and academics that challenge the very boundaries of what counts as science, evidence, and historical narrative.

The article proposes a methodological shift: from treating Indigenous knowledge systems as objects of analysis to engaging with them as sources of conceptual insight. Drawing from interdisciplinary debates across anthropology, Indigenous studies, museum studies, and the history of science, it argues for an expanded evidentiary repertoire – one that recognizes oral narratives, ritual practices, and relational ontologies as valid and generative forms of knowledge.^9^ Rather than merely enriching existing frameworks, this approach reconfigures the very terms of inquiry, allowing Amazonian case studies to illuminate broader tensions within the history of science. It requires historians of science to confront the extractive logics embedded in our own archives and to consider how sustained engagement with other epistemologies can reshape the field from within.

In this article, I argue that Amazonian artifacts collected during colonial scientific expeditions hold significant potential to advance our understanding of the relationship between Indigenous knowledge systems and Western science, provided they are approached critically and relationally. That is, rather than treating these objects as isolated entities, a relational approach considers the networks of interaction, affect, and reciprocity through which they acquire meaning – whether in ritual, kinship, or cosmological contexts.^10^ These objects are not passive carriers of meaning; they are entangled in systems of interpretation, appropriation, and resistance.^11^ Addressing them demands a methodological openness to nontextual sources and a willingness to question the disciplinary boundaries that have historically shaped knowledge production.

To illustrate this argument, I turn to distinct yet interrelated sets of Amazonian artifacts currently held in European institutions: a constellation of ethnographic objects exhibited behind glass at the University of Coimbra – originally assembled by the Portuguese naturalist Alexandre Rodrigues Ferreira during his *Viagem Filosófica* (1783–92) – and botanical specimens collected by the English naturalist Richard Spruce in the nineteenth century, now preserved at the Royal Botanic Gardens, Kew. Rather than centering the analysis on a single object, my focus lies on the aforementioned curatorial ensemble in Coimbra’s “Cabinet of Curiosities” in which Jurupixuna masks are displayed alongside phrenological casts, zoological specimens, and African statuary, all devoid of explanatory context. Assembled in the course of Ferreira’s imperial expedition, Coimbra’s Amazonian collection stands today as one of the most extensive and significant holdings of such material outside the region, and constitutes a critical site for interrogating the entangled histories of scientific exploration, imperial ambition, and Indigenous material worlds. Together, these two configurations form what I term a cosmo–historical juxtaposition – a heuristic device that brings into sharp relief not only the contrasting imperial formations (Portuguese and British) and temporalities of extraction, but also the entangled Indigenous ontologies embedded in the artifacts, grounded in themes such as human–animal transformation and vegetal–human relationality.

This approach is informed by recent collaborations with Indigenous artists and intellectuals, whose engagements with museum collections reveal cosmological dimensions that remain invisible to traditional exhibitions.^12^ These reinterpretations challenge the authority of curatorial narratives and highlight the ongoing vitality of the objects themselves – as vessels of ancestral presence, as repositories of memory, and as agents of knowledge transmission.^13^ They prompt us to ask: what happens when objects taken as “evidence” in past scientific expeditions return, not physically but conceptually, through contemporary Indigenous frameworks of interpretation? What kind of history emerges when we allow myth, dream, and ritual to coexist with archival documentation and material analysis?

Indigenous thinkers such as Yanomami shaman Davi Kopenawa have made this challenge explicit. In *The Falling Sky*, Kopenawa denounces museums as sites of cosmological captivity, describing the objects they contain not as inert artifacts but as living presences, torn from their communities and trapped in foreign epistemologies.^14^ From this perspective, the museum is not only a place of collection but also of rupture – a site where the relational worlds of the objects are suspended. Kopenawa’s critique, alongside that of other Amazonian intellectuals, demands that we take seriously the ontological and ethical implications of working with such materials. It also urges us to recognize the limits of Western analytical categories and the need to envisage alternative frameworks.^15^

In dialogue with these perspectives, I draw on Eduardo Viveiros de Castro’s notion of “counter-anthropology,”^16^ and broader theoretical engagements with the occult or relational life of things, as developed by Fernando Santos Granero and others.^17^ These approaches invite a reconfiguration of the epistemic status of Amazonian artifacts – not as inert objects of study, but as conceptual agents that challenge the assumptions of Western epistemology. Counter-anthropology, rooted in Amerindian perspectivism, shifts the analytical locus from viewing Indigenous thought as a mere object of representation to engaging it as a mode of theorization in its own right.^18^ Within this framework, masks and plants are not merely ethnographic or natural history specimens: they are vital expressions of ontological worlds in which the boundaries between human and nonhuman, nature and culture, presence and evidence are radically fluid and continually negotiated.^19^

Ultimately, this article is not about recovering an Indigenous past in the service of Western historiography. It is about reorienting the field of the history of science to reckon with ontological pluralism, epistemic injustice, and the material afterlives of colonial extraction.^20^ While drawing conceptually on Indigenous and anthropological theory, its analytical movement is anchored in historical inquiry. Rather than applying abstract ontological frameworks to empirical cases, I trace how specific collections have historically produced and sustained epistemic asymmetries. At the same time, I ask how the tools of historical analysis – attuned to archival silences, material traces, and the shifting contours of actor-worlds – might also contribute to decolonial theory. The dialogue between history and Indigenous thought is not unidirectional: it opens up space for methodological renewal and conceptual critique within the history of science itself. By treating Amazonian artifacts as dynamic sites of entanglement – between empire and Amerindian metaphysics, between scientific ambition and Indigenous sovereignty – I aim to contribute to broader conversations about the decolonization of knowledge, the ethics of display, and the transformative potential of Indigenous epistemologies in reshaping how we write the history of science.

## The transit of Amazonian artifacts and the role of Indigenous labor

The transit of Amazonian artifacts to the Global North during the eighteenth and nineteenth centuries represents a multifaceted historical phenomenon, intricately tied to European scientific exploration, colonial expansion, and anthropological inquiry.^21^ At the center of this dynamic was the extensive use of Indigenous labor, which played a foundational role in the surveying of the colonial Amazon and the collection of natural history materials.^22^ Indigenous people were primarily mobilized in two critical areas: river navigation and the extraction of commodities known as *Drogas do Sertão* (Amazonian spices), including cacao, Brazil nuts, *guaraná*, *pau-cravo*, *bixa orellana*, and *sarsaparilla*.^23^ These goods fed into a growing early modern global market, where their value rose as a result of European consumer demand.^24^ Recent studies have emphasized not only the material but also the epistemic centrality of Indigenous ecological knowledge in these processes.^25^

Historians have shown that Jesuits were instrumental in the early cultivation and commercial exploitation of cacao, transforming it into a lucrative export for the Portuguese Crown, in particular with the growth of chocolate consumption in Europe.^26^ By the 1680s, the Amazonian colonial economy was simultaneously expanding through agriculture and extraction. Agricultural frontiers pushed toward areas near Belém, especially the Tocantins River Valley, where sugar, coffee, cacao, cotton, beans, and manioc were cultivated. Meanwhile, intensive extractive practices were unfolding in the *sertões* (hinterlands), targeting wild cacao, clove bark, *sarsaparilla*, and *copaíba* oil.^27^ This strategic shift from coastal monocultures to forest extraction was driven in part by Portugal’s declining control over East Asian spice routes and the rising value of Amazonian commodities in European markets.^28^

Throughout much of the seventeenth century, slavery remained legal in the region, and the exploitation of these forest products was deeply entangled with the Indigenous slave trade.^29^ Legal frameworks for Indigenous enslavement included ransoming (*resgates*) and the so-called just wars. Free Indigenous individuals were frequently relocated from the *sertões* to missionary villages (*descimentos*), where they were compelled to labor for both settlers and the Crown. While the legal and institutional contours of Indigenous labor changed over time, its centrality to colonial economic and scientific projects remained consistent – generating ongoing tensions between missionaries, settlers, and civil authorities.^30^ Crucially, these enslaved and coerced individuals carried not only goods but also knowledge: Their navigational skill, ecological expertise, and labor were woven into the very fabric of colonial science.

Canoes were indispensable to mobility and extraction.^31^ By the mid-seventeenth century, Jesuits relied on canoes to travel upriver in search of Indigenous captives held by rival groups. Rather than using force, they often secured these individuals through trade-based ransoms – ensuring access to labor without open conflict. The indispensability of canoes and Indigenous botanical knowledge made spice-collecting expeditions a natural extension of preexisting trade networks. In effect, Indigenous expertise – ranging from navigation and riverine logistics to botanical identification and ecological knowledge – underwrote both the extractive economy and the accumulation of natural knowledge. Together, these skills formed an unacknowledged epistemic infrastructure for European natural history.^32^

The Jesuit João Daniel, who resided in the Portuguese Amazon for an extended period, underscored the social and technical significance of Indigenous pilots, describing piloting as both a skilled profession and a form of art. Within Indigenous settlements, he observed, this role constituted one of the most esteemed positions, with pilots commanding considerable respect and authority among community members.^33^ These Indigenous navigators earned their reputations by guiding military and scientific expeditions through vast and often unmapped territories: collecting Amazonian clove in the forests of the Xingu River, cacao along the Madeira and Solimões, turtles and their eggs on Amazonian beaches, and *sarsaparilla* in the swampy lowlands of the Jari. Their expertise – local, embodied, and accumulated over generations – was instrumental to the formation of European scientific collections from the eighteenth to the twentieth century.^34^

Naturalists such as Alexandre Rodrigues Ferreira, Carl Friedrich von Martius, Johann Natterer, Alfred Wallace, and Richard Spruce depended extensively on Indigenous labor and logistical networks – not only to access remote regions and ensure their mobility, but also for the collection, preparation, preservation, and transport of botanical and ethnographic materials.^35^ The scientific investigations carried out during these expeditions yielded a diverse array of objects, ranging from ceremonial masks and featherwork to utilitarian tools and textiles. These artifacts offered European scholars tangible insights into the lifeways of Indigenous groups such as the Munduruku, Jurupixuna, and Yanomami, among many others.^36^ Documentation practices varied considerably: some objects were carefully recorded through field notes, illustrations, and correspondence, while others were acquired through coercion or outright appropriation. This disparity highlights the deep asymmetries of power that shaped the colonial production of scientific knowledge.

Once transported to Europe, these objects were incorporated into museum collections, where they were typically displayed as “curiosities” or relics of primitive societies – interpretative frames that emphasized their exoticism and supported narratives of European civilizational superiority.^37^ European institutions such as the Royal Botanic Gardens, Kew, the Science Museum of the University of Coimbra, and others played critical roles in shaping the ways in which these materials were classified, preserved, and publicly displayed, thus contributing to the epistemological frameworks through which colonial natural history was constructed and legitimized.^38^ These practices not only cemented Western epistemic authority, but also erased or distorted the Indigenous labor and knowledge that had made these collections possible in the first place.^39^

## Science on display: glass cabinets, colonial knowledge, and Amazonian artifacts

It is precisely within this entangled legacy of epistemic extraction and representational control that the collections of the Science Museum of the University of Coimbra demand closer attention. Far from incidental, the selection of this particular assemblage reflects both the scale of Portuguese imperial ambition and the institutional centrality of Coimbra in the history of colonial science. The museum houses one of the most extensive and prominent Amazonian collections outside the Amazon itself.^40^ This collection began taking on renewed significance and visibility in Brazilian academic and public discourses in 1997 when a portion thereof was temporarily returned to Brazil to commemorate the 500th anniversary of Portuguese colonization.^41^ Coimbra occupies a central place in the scientific and imperial infrastructures of the Portuguese Enlightenment. In the mid-eighteenth century, under the direction of the Marquis of Pombal, the University underwent sweeping reforms aimed at modernizing education and strengthening the colonial apparatus through science.^42^ It was within this context that Domenico Vandelli – a Venetian naturalist and key figure in the modernization of Portuguese science – was brought to Coimbra to teach natural philosophy and to design an empirical curriculum aligned with Enlightenment ideals.^43^ From this institutional reform emerged a new generation of naturalists, among whom Alexandre Rodrigues Ferreira stands out. Between 1783 and 1792, Ferreira undertook the *Viagem Filosófica* – one of the Portuguese Crown’s most ambitious scientific expeditions to its overseas territories.^44^ The collection that resulted from his travels through the Amazon – comprising botanical, zoological, mineral, and ethnographical materials – was destined to furnish the newly founded cabinets of natural history in Coimbra and Lisbon, and continues to shape museum collections to this day.

**Figure 1.**
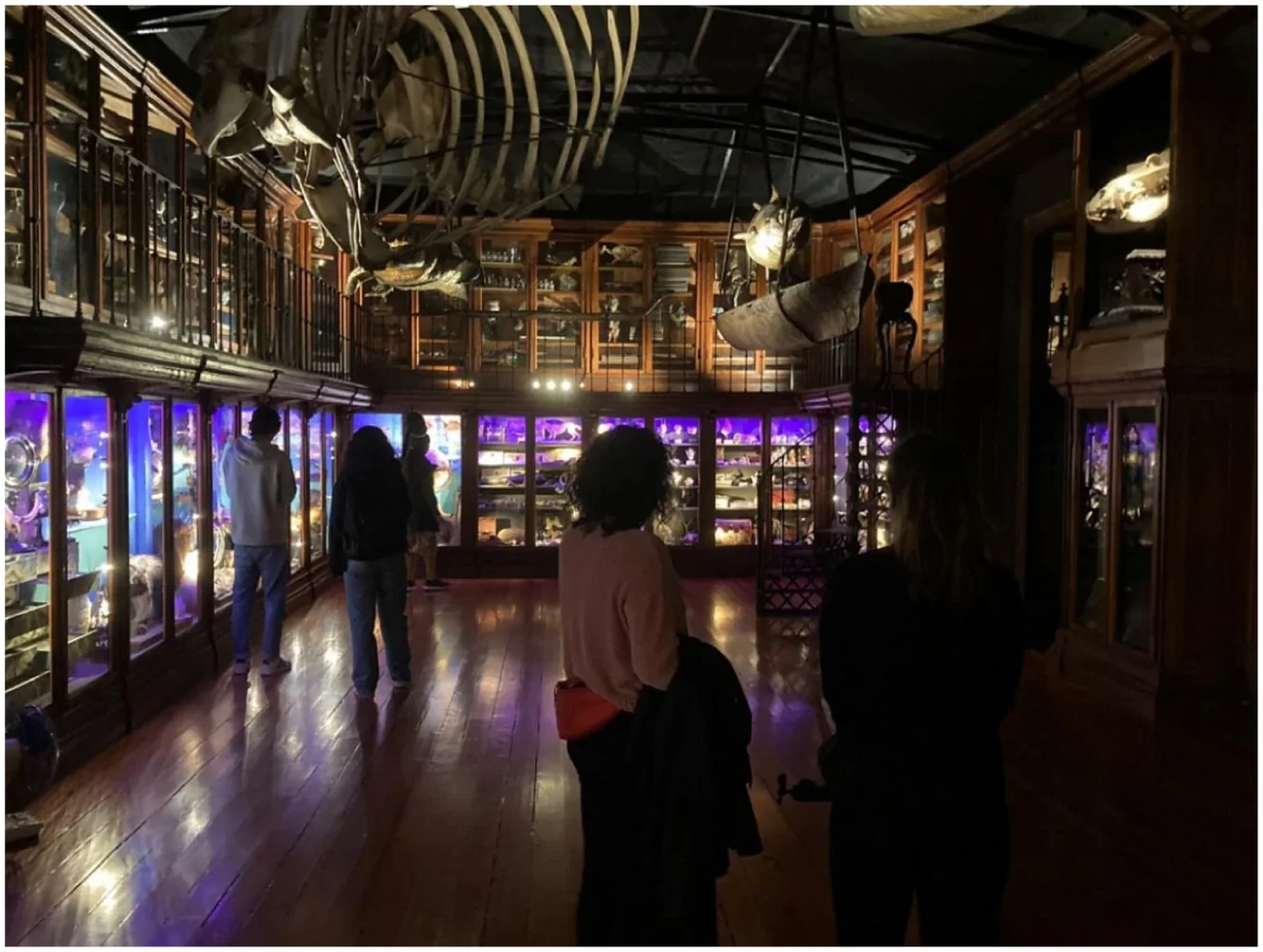
Image taken at the “Cabinet of Curiosities” exhibition at the Science Museum of the University of Coimbra, 2023. Author’s photo.

**Figures 2 and 3.**
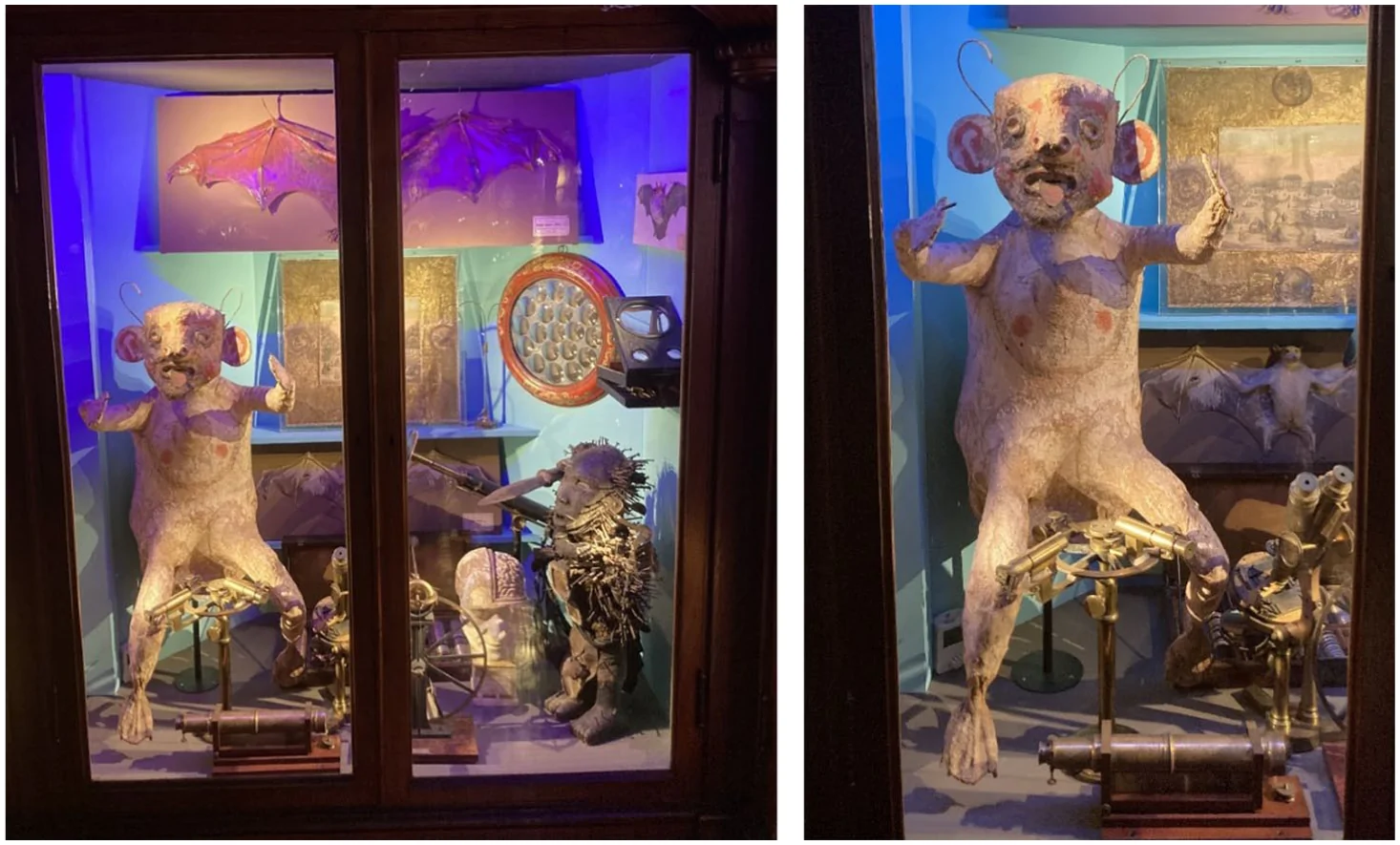
The “Cabinet of Curiosities” exhibition at the Science Museum of the University of Coimbra, 2023. Author’s photo.

I first encountered the University of Coimbra’s Science Museum several years ago in passing, but it was during a return visit in 2022 that the weight of its collections – and the silences they carry – truly revealed themselves. As a historian of science, this renewed engagement offered a lens through which to observe how the Portuguese state continues to mobilize science and memory to project power and cultural authority. As someone born and raised in the Brazilian Amazon, I was equally struck by the decontextualized manner in which the artifacts – many of which originate from my region – were arranged in the museum’s permanent exhibition. The “Cabinet of Curiosities” exhibition invites visitors to “experience a whole world made up of unusual and beautiful objects.”^45^ Here, artifacts are displayed behind glass – glass that isolates, fragments, and objectifies ( Figures 1–3). The display brings together objects from vastly different times and places – ritual pieces, scientific instruments, and Indigenous materials – without any explanatory context. Presented as curiosities, they appear suspended from the histories and cosmologies that once gave them meaning.

This mode of display – through transparent vitrines that promise visibility while simultaneously enforcing epistemic distance – echoes the logic of early modern science, which sought to produce knowledge by isolating and classifying objects removed from their original contexts.^46^ As scholars such as Sharon Macdonald, Tony Bennett, and James Clifford have noted, such exhibitions aestheticize cultural difference while reinscribing colonial hierarchies of knowledge, presenting Western modes of seeing as universally valid.^47^ Despite recent shifts in museological discourse, many critical efforts remain limited when it comes to ethnographic artifacts from the Global South. In Coimbra’s exhibition, the Amazon and Africa are conflated both spatially and symbolically, reproducing a long-standing imperial imaginary in which these regions were framed as interchangeable frontiers – raw material zones for scientific extraction and imperial desire. By placing Indigenous and African artifacts alongside European scientific instruments without contextual mediation, the display collapses epistemic distinctions – conflating objects of inquiry with tools of knowledge-making, and further reinforcing this imperial vision.

One striking example is a zoomorphic mask crafted from bark and brought to Europe by Alexandre Rodrigues Ferreira during his *Viagem Filosófica*. Part jaguar, part monkey, the mask was originally part of a larger set attributed to the Jurupixuna peoples of the Rio Negro. In the glass display, however, the mask shares its case with optical instruments, preserved animals, and racialized human remains – further blurring distinctions between categories of life and modes of knowing. Ferreira’s journey aimed to collect and classify both nature and culture as an imperial resource. The resulting collection, now housed in the museum’s reserves and galleries, represents one of the largest assemblages of Amazonian artifacts in Europe.^48^

Yet beyond the historical narrative of Enlightenment science and colonial classification, what remains underexamined is how these artifacts – housed in vitrines that both protect and silence – retain traces of cosmologies that resist their assimilation into Western taxonomies. These vitrines not only render Indigenous knowledge invisible; they also enshrine an illusion of neutrality and authority. The juxtaposition of botanical specimens, ethnographic artifacts, and scientific instruments masks the epistemic operations of extraction – processes through which colonial science appropriated Indigenous worlds while erasing the ontologies that once animated them. It is precisely in response to this kind of epistemic enclosure that Indigenous intellectuals such as Davi Kopenawa have articulated critiques – not just of colonialism, but of the ontological violence perpetrated by institutions of collection and display.^49^

From a Kopenawian perspective, masks such as the one exhibited in Coimbra are not simply objects or representations; they are animated forms that condense relations between humans, animals, and spirits.^50^ Kopenawa’s *The Falling Sky* describes a cosmos in which the boundaries between human and animal are porous, and where transformation is not metaphorical, but ontological. Spirits, or *xapiri*, often take animal form – jaguars, monkeys, birds – and it is through these forms that they dance, communicate, and manifest in the visions of shamans.^51^ The presence of zoomorphic forms in ritual contexts is not aesthetic but relational: they are part of a world in which beings transform, become visible, and speak. Displayed in vitrines, these beings are immobilized. Their isolation behind glass reflects the interruption of the dialogical relation that sustains their vitality and intelligibility.^52^ The mask behind glass becomes not just an artifact, but a silenced interlocutor – an image of a spirit deprived of its voice.

For Kopenawa, the act of looking – especially when unaccompanied by ritual knowledge – is never neutral. The *napë* (white people) are described as those who see without understanding, who desire images but ignore the depth of the forest’s living knowledge.^53^ In this light, the scientific display of zoomorphic artifacts reveals more about the epistemology of the museum than about the worlds from which these objects emerge. It transforms living beings into “image-skins” (*utupë*) detached from their original paths, much like the *xapiri* dancers lose their shine when their mirrors are broken.^54^ A Kopenawian reading of the museum vitrines thus invites a reversal: to look not at the mask as an object of curiosity, but to understand how the mask might be looking back – assessing the gaze of those who claim to know. It demands not merely representation, but relation; not interpretation, but cosmopolitical accountability.

## An Amazonian’s cosmology: the Yanomami critique

What happens when taxidermied animals, dissected plants, and ethnographic artifacts housed in natural history museums across the Global North are analyzed not through classificatory or representational logics, but through the conceptual lens of Indigenous cosmologies? Grappling with this question requires more than a critique of museological display or a call for institutional inclusion. It demands a sustained engagement with the ontological and epistemological frameworks of the communities from which these objects were extracted – frameworks that have long been muted or distorted within institutions shaped by colonial and scientific authority. Rather than treating these artifacts as passive residues of a distant cultural past, this perspective invites us to consider them as cosmopolitical agents – entities embedded in living systems of knowledge, obligation, and transformation.^55^

This section returns to the thought of Davi Kopenawa, the Yanomami shaman and intellectual whose work offers one of the most incisive critiques of extractivism in both its material and epistemic forms. His cosmopolitical vision challenges not only the practices of collection and display, but the very assumptions underlying Western notions of objectivity, evidence, and knowledge. Born around 1956 in the village of Marakanã, near the Brazil–Venezuela border, Kopenawa’s life embodies the entanglements of Indigenous and non-Indigenous worlds. After losing much of his family to epidemics triggered by incursions from state agents and missionaries, he began working as a translator for Brazil’s National Indian Foundation (FUNAI) while still a teenager. Yet Kopenawa’s act of translation has always extended far beyond language. It constitutes a philosophical and cosmological mediation – a sustained attempt to make the Yanomami world intelligible without subjecting it to the classificatory violence of Western epistemologies.^56^

The term “Yanomami” itself – *yanõmami thëpë*, meaning “human beings” – articulates a fundamental relational ontology. The forest (*urihi*) is not an inert backdrop, but a living, sentient space endowed with breath (*wixia*), spirit (*në rope*), and vital images (*utupë*).^57^ Animals, plants, stones, rivers, and winds are not resources or representations, but participants in an ongoing cosmological drama. Shamans, by cultivating relationships with *xapiripë* – miniature spirit-beings who manifest as ancestral images – sustain this balance. These practices are not metaphorical. They constitute a mode of knowledge, care, and diplomacy in a world where ontological boundaries between human and nonhuman, nature and culture, are not presumed but continually negotiated. This relational orientation is what many Indigenous thinkers describe as cosmopolitics – a way of engaging with the world in which knowledge is not merely a representation of reality, but a vital, living practice inseparable from the ethical, spiritual, and ecological relations that sustain life.^58^

Kopenawa’s critique becomes particularly incisive when brought to bear on exhibitions such as “Cabinet of Curiosities” at the Science Museum of the University of Coimbra that displays Amazonian ritual objects alongside remnants of imperial science and racial taxonomy. In one exhibition case, a Jurupixuna mask is juxtaposed with optical instruments, a phrenological cast, a preserved bat specimen, and a Niksi statue from the former Portuguese colonies in Africa, likely Angola. Until recently, the display also included mummified heads of Munduruku individuals and skulls from African populations – still housed today in the museum’s technical reserve. These juxtapositions – stripped of provenance and explanatory context – reproduce colonial taxonomies in which objects are abstracted from the lifeworlds that gave them meaning.^59^ From Kopenawa’s perspective, however, this is not merely a failure of representation; it is an ontological and cosmopolitical rupture. Masks and other Amazonian artifacts are not inert matter but vital extensions of the *urihi*. Severed from their cosmological networks and displayed under classificatory logics foreign to their world, these objects become unstable presences – capable of generating harm or revealing the violence that made their displacement possible.^60^

In this sense, Kopenawa reframes museum critique in radical terms. His thought compels historians of science to go beyond questions of access, inclusion, or provenance, and to reckon with the cosmologies that such institutions systematically efface. Rather than viewing museums as neutral repositories of knowledge, Kopenawa invites us to see them as sites of ontological friction, where divergent worlds – and their material embodiments – collide.^61^ His intervention thus opens up the possibility of reimagining the museum not only as a space for critical reflection but as a terrain for epistemic repair and cosmopolitical negotiation.

## Subjectivity, dreams as ways of knowing, and a new politics of display

Within the historiography of science, scholarly inquiries that concentrate on material culture originating in the Global South frequently center their analyses on the transformative processes these objects have undergone under imperial frameworks and within colonial networks of knowledge production.^62^ While such an emphasis represents a significant advance in understanding the historical trajectories of these artifacts, substances, and the references attached to them, the cosmological worldviews and epistemological notions that shaped their genesis and original meaning remain to be fully incorporated into the explanatory frameworks of the history of science.

This section examines how such epistemologies – particularly the Yanomami worldview articulated by Davi Kopenawa – offer not merely ethical or political critiques, but robust conceptual challenges to dominant notions of evidence, memory, and subjectivity in the history of science. In particular, I ask, how might assertions about dream-knowledge and spirit-agency be placed in dialogue with the long history of what counts as proof and evidence in scientific traditions?^63^ What historiographical tools might allow us to treat these claims not as symbolic metaphors, but as part of a broader and contested field of evidentiary production?^64^

Kopenawa defines the museum as “a place where the traces of the ancestors of the forest dwellers who have been gone for a long time are kept locked away.”^65^ In these institutions, he recognizes a multitude of Indigenous artifacts taken from the Amazon – ceramics, baskets, bows, arrows, flutes, feather ornaments – objects collected by naturalists and anthropologists over the centuries as part of scientific expeditions to South America.^66^ He describes them not as inert remnants but as living entities imbued with ancestral presence. According to Kopenawa, these are “very old possessions,” some of which belonged “to great shamans who died a long time ago.”^67^ Their materiality cannot be separated from the cosmological systems through which they were made, used, and understood.

In contrast to dominant epistemologies that ground knowledge in textual inscription, replicability, and disembodied observation, Kopenawa asserts a form of evidentiary reasoning anchored in spiritual continuity, intergenerational memory, and dream-based communication. For the Yanomami, these objects hold captive the images of the deceased – images that can no longer “come to dance,” that is, can no longer mediate knowledge transmission between shamans and spirits.^68^ “Their paths to us were cut off too long ago,” he laments.^69^ Here, objects are not only residues of past practices but active agents whose dislocation from their cosmological circuits produces epistemic rupture. This rupture translates into a transformation of meaning: masks become inert displays, plants become depersonalized specimens. What is lost is not only context but cosmological function and intersubjective relation. This notion resonates with what Lorraine Daston and Peter Galison have described as the history of “epistemic virtues” – forms of moralized epistemic conduct historically associated with scientific objectivity – yet it emerges from a radically different ontological ground: one in which witnessing, authority, and transmission are not rooted in detachment or neutrality, but in relational obligations to nonhuman entities, including spirits, forest beings, and ancestral images.^70^

Crucially, Kopenawa ties the efficacy of knowledge to the practice of dreaming. “White people no longer know how to dream with the spirits,” he asserts, situating dreams not as private symbols but as public, actionable events within a larger cosmological order.^71^ Dreams – especially those induced by *yãkoana* and mediated by *xapiri* spirits – function as a mode of cognition and epistemic disclosures.^72^ The following quote encapsulates this logic:

The shamans, as I said, do not sleep like other men . . . At night, however, the xapiri continue to sing to them in the time of dreams . . . This is how shamans manage to dream of the devastated lands surrounding our forest and the boiling epidemic fumes that arise from them. Only the xapiri truly make us wise, because when they dance for us, their images expand our thoughts.^73^

In this passage, knowledge of environmental degradation, epidemic causality, and spiritual imbalance is not derived from empirical measurement but from dream-induced relations with spirit-beings. If we consider Steven Shapin’s analysis of “invisible witnesses” in early modern science – where trust in knowledge production depended on reputation rather than direct observation – or recent science and technology studies (STS) work on affective and embodied epistemologies, Kopenawa’s dream-epistemology compels us to further widen the historiographical frame.^74^ It asks us not only to take seriously nontextual, relational modes of knowledge, but also to engage with ontologies in which presence, truth, and verification are grounded in radically different metaphysical commitments – ones based on intersubjective bonds with nonhuman entities, rather than detachment from them.

His critique of museums, then, is not merely moral; it questions the epistemological assumptions that underpin exhibition practices. While he acknowledges that exhibitions can serve positive political ends – such as protecting the forest – he draws a sharp distinction between displaying “our images” and exhibiting “things that belong to ghosts.”^75^ His horror at the sight of corpses in vitrines, “like children with wrinkled skin,” draws attention to the affective and ontological violence enacted by scientific collection practices.^76^ What Kopenawa exposes, from within an entirely different ontological register, are the affective costs and metaphysical presumptions embedded in such rationales.

A more robust historiographical engagement would thus juxtapose his cosmopolitical denunciation with the archival traces of nineteenth-century figures like Carl von Martius, or Richard Spruce, whose journals often frame Indigenous artifacts as “disappearing evidence” of a doomed race – an impulse that simultaneously laments and justifies their extraction.^77^

What Kopenawa exposes, in short, is the lingering contradiction in the history of science’s evidentiary regime: that objects of proof were often obtained through acts of dispossession; that knowledge was built upon the estrangement of meaning from objects torn from their epistemic lifeworlds. His observation – “Do they want to get them in anticipation of our death?” – is both metaphor and methodology.^78^ It forces a confrontation with the assumptions underlying collection, preservation, and display as scientific practices. This, I argue, is where the intersection between Kopenawa’s critique and the history of science becomes most productive: in the demand that we historicize our own evidentiary norms – not as neutral standards, but as situated, extractive, and open to radical reinterpretation. Echoing scholars such as Donna Haraway, Ann Laura Stoler, and Michel-Rolph Trouillot, this approach calls for a critical reexamination of the epistemic foundations of the archive, the museum, and the very category of evidence itself.^79^

By framing shamanic dreams and cosmopolitical agency not as cultural beliefs but as alternative ways of producing and verifying knowledge, Kopenawa compels us to expand what counts as legitimate epistemological labor. His perspective, while grounded in a particular Amazonian world, raises broader historiographical implications: can the history of science accommodate forms of knowing that do not aspire to universality, falsifiability, or textual stabilization? Or must it redefine its methodological coordinates to remain accountable to the plurality of ways through which humans – and more-than-humans – have sought to engage with, interpret, and transform the world?

These questions take on renewed significance when we turn from cosmological critique to the material itineraries of plants themselves – particularly those collected in the name of science and empire. One such itinerary begins with the nineteenth-century expeditions of Richard Spruce in the Rio Negro region, whose botanical fragments, now preserved at Kew Gardens, carry the layered afterlives of both imperial extraction and Indigenous entanglement.

## Richard Spruce, the Rio Negro, and the colonial afterlives of plant fragments

**Figure 4.**
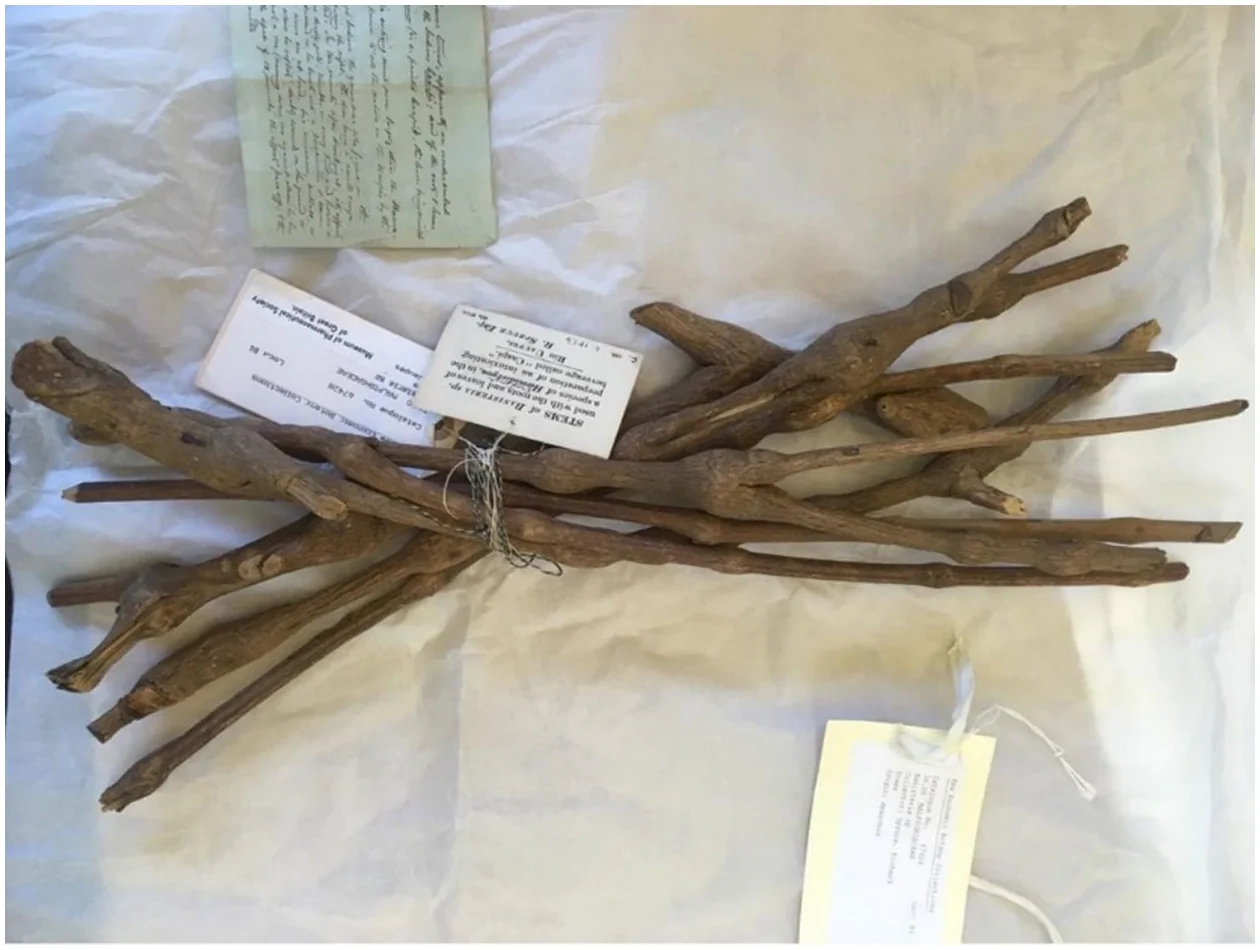
Image taken at the Museum of Economic Botany at the Royal Botanic Gardens, Kew, 2020. Author’s photo.

My encounter with the plant fragments (Figure 4) now housed at the Royal Botanic Gardens, Kew, began in 2020, during a research visit focused on Amazonian botanical specimens in European collections. Among these, I was drawn in particular to the materials collected in the Rio Negro region by English botanist Richard Spruce in the mid-nineteenth century. The convergence of Kew Gardens, the Rio Negro, and Spruce himself emerged as a compelling triad for interrogating the entangled histories of colonial science. What began as my own empirical inquiry into specimen provenance soon unfolded into a reflection on the epistemic dislocations and cosmological erasures embedded in these collections – particularly when considered from the standpoint of the Indigenous peoples of the region.

Spruce’s collection is one among thousands of Amazonian specimens dispatched to Europe by nineteenth-century naturalists such as Spruce, Wallace, and Bates. Inspired by the legacy of figures like Carl von Martius and Alexander von Humboldt, these explorers entered the Amazon at a time when Brazil, newly independent from Portugal, had opened its ports to European powers.^80^ Britain, in particular, benefited from this access, establishing deep economic and geopolitical ties with Brazil.^81^ Historian Leslie Bethell notes that by the 1820s, Brazil had become the third-largest market for British textiles and goods, with the city of London serving as a central node for investment in Latin American infrastructure, agriculture, and extractive industries.^82^ This period marked the Amazon’s increasing integration into global circuits of commerce, extraction, and scientific knowledge production.^83^

Within this context, Spruce embarked on his journey in June 1849. Over fifteen years, he traveled through Brazil, Venezuela, Peru, and Ecuador, charged with collecting herbarium specimens for Kew Gardens.^84^ His departure coincided with the founding of the Museum of Economic Botany at Kew by William Hooker, designed to curate specimens of “useful plants” for redistribution throughout the British Empire.^85^ Spruce’s role in this network was foundational: he collected hundreds of specimens, including *sarsaparilla*, *cinchona*, and *Hevea brasiliensis*, plants already familiar to European science via colonial encounters in the Caribbean and Guiana. His work also reflected a deeper imperial ambition – to map, classify, and appropriate the Amazon’s biodiversity as part of Britain’s expanding natural history archive.^86^

Spruce’s letters to George Bentham and William Hooker document the scope of his contributions. Between 1851 and 1853, he identified and shipped at least 195 natural history items, including seeds, plant specimens, and ethnographic objects. In a letter from San Carlos do Rio Negro dated June 27, 1853, Spruce described a “small collection for your museum,” including guaraná seeds, palm fruits, cashew wood, arrows, and rings made of tucumã kernel.^87^ These objects were gathered during his time in the Vaupés region and along the Rio Negro, where Spruce also made his first recorded mention of *Banisteriopsis caapi*, the plant used to prepare the psychoactive brew *ayahuasca*. In describing a feast he witnessed in 1852, known as *dabucuri*, he remarked on the presence of *caapi*, *caxiri*, *tobacco*, and *paxiuba flutes* – musical instruments used in sacred rituals. Spruce would later smuggle four of these flutes onto a ship bound for Venezuela, wrapping them in mats and hiding them under the floorboards to avoid detection by his Indigenous crew.^88^

This moment – documented in his correspondence – is telling. It reveals not only the extractivist logic behind the acquisition of ethnobotanical materials but also the deliberate rupture of cosmological relations. The sacred instruments, the plant, the feast: all were fragmented, disembedded from their relational context, and shipped to Europe as isolated specimens. Like many naturalist-travelers, Spruce transmuted Indigenous ontologies into botanical data, reducing cosmologically charged entities to museum objects.

Spruce’s logistical routes and labor systems relied on infrastructures built through centuries of evangelization and colonial expansion – Jesuit missions, Salesian networks, regional elites, and enslaved Indigenous workers. His expedition was no exception. In his letters, Spruce referred to the Indigenous guides and collectors he employed as “my Indians,” implicitly disclosing the dependency of his enterprise on their expertise.^89^ They guided the expedition through rivers and forest trails, and identified and prepared botanical specimens with care – labor rooted in generational knowledge. The knowledge they embodied – often gained through intergenerational transmission – was fundamental to the success of his expedition, even as it was routinely rendered invisible in scientific accounts.

Moreover, Spruce’s collection took place in a region that was undergoing intense transformation. Following the opening of Amazonian ports, the basin became a site of feverish resource-driven activity, with immigrants, traders, and scientists competing for access to its resources. The northwestern Amazon saw the imposition of slave labor systems, including those orchestrated by the Portuguese Crown and later administered through Salesian missions.^90^ These systems conscripted Indigenous labor into the service of empire: constructing riverside cities, maintaining logistical networks, and supporting the scientific missions that fed collections like Kew’s.^91^ In this sense, Spruce’s specimens are not only botanical; they are archival fragments of a larger colonial apparatus, shaped as much by geopolitical forces as by the hands that gathered them.

Today, these materials continue to sit in herbarium cabinets and museum displays, more often than not presented as neutral scientific specimens. Their afterlives suggest otherwise, however. The stories of their collection – of hidden flutes, smuggled seeds, and dislocated plants – reveal a palimpsest of erasure, survival, and contested meaning. Importantly, the Indigenous thinkers whose voices guide this analysis – such as Jaime Diakara and João Paulo Lima Barreto – speak out from within genealogies shaped by the lived and inherited experience of colonial and missionary violence. The epistemologies they articulate are not abstract systems but living responses to historical rupture: intellectual practices that emerge in spite of – and often against – the forces that sought to silence them. Rather than seeking mere recovery, a decolonized approach to the history of science and knowledge demands that we center these perspectives not as marginal contributions, but as foundational to any serious account of how knowledge has been produced, contested, and sustained across worlds. This means rethinking not only the histories of the objects themselves, but the conceptual frameworks through which they are interpreted.

## The vegetal world through the Tukano cosmology

Following this imperative to center Indigenous cosmologies and the epistemologies forged in the wake of colonial violence, this section turns to a different kind of artifact: plant fragments. It revisits the *caapi* specimens collected by the English botanist Richard Spruce in the mid-nineteenth century now housed at the Royal Botanic Gardens, Kew. To engage meaningfully with these materials, one must consider not only their botanical taxonomy as a residue of colonial science, but also the cosmological terrain from which they emerged.

When Spruce traveled through the Rio Negro, he moved through the ancestral territories of the Tukano peoples – one of over four hundred Indigenous groups who have long inhabited, and continue to inhabit, the Amazon Basin. Contemporary ethnographic and Indigenous scholarship has illuminated the intricate webs of relation that bind plants, animals, and humans in this region, revealing a world not organized by classification, but by transformation and kinship.^92^ Among these contributions, the work of Tukano researcher Jaime Diakara is particularly vital. Drawing from ancestral memory and oral history, Diakara recounts a time “before time,” when *Caapi* (*ayahuasca*) first appeared not as a plant, but as a human child.^93^ According to this narrative, *Caapi* – also known as *Gaapi* – was born at the threshold of time, coinciding with the emergence of the Tukano peoples in the Vaupés region. Its human form was dispersed, its body divided among the founding families of the region, making the plant not only a living being but a genealogical link. To consume *Caapi* is thus never merely to ingest a substance; it is to enter into a relation with a primordial being, to activate a mode of knowledge transmission that exceeds both language and lineage.^94^

The myth unfolds with the arrival of the first men of the world, who descended upon a colorful and glowing primordial serpent canoe. Upon landing in the forest, these men built the first hut, or “maloca,” where they drank cashiri and other intoxicants while awaiting a promised gift from Father Sun. Inside the maloca, the Sun created the first woman, called Yagé-Woman, already pregnant due to the Sun’s gaze. During labor, Yagé-Woman walked in the forest until nightfall, giving birth to her Yagé (*ayahuasca*) vine son, whose light guided her back to the maloca. Holding the baby-vine, Yagé-Woman asked, “Who is the father of this vine-boy?” One man bravely claimed paternity, cutting a piece of the umbilical cord; others followed, and in this way, each Amazonian Indigenous group received a piece of the Sun’s gift.^95^

Understanding the Tukano perspective on the “vegetal world” requires engaging with conceptual frames such as so-called Amerindian perspectivism.^96^ This cosmological philosophy originates from the worldview and cultural practices of Indigenous peoples in the Amazon region. It offers a unique understanding of their reality, positing that the world is inhabited by diverse beings or “persons,” each possessing their own subjectivity and perspective. These beings include not only humans but also animals, plants, and inanimate objects, each with consciousness and agency.^97^ In this context, *caapi* plants exemplify subjectivity, representing human attributes. The Tukano view holds that even after being transported across the Atlantic, the plant maintains its ontological status as an animate entity. In Amazonian ontologies, things – at least some things – are considered as possessing social life.^98^

The social life of things in Amazonian thought extends beyond their movement through different “regimes of value,” as proposed by Arjun Appadurai, to encompass the ways humans and things relate as subjectivities.^99^ In Native Amazonian thought, the formation of life is a constructive process in which primordial bodies and body parts play a crucial role. Artifacts were understood to emerge from transformations of persons or their body parts, later taking the form of other beings. The myth of the *Caapi* baby’s birth implies that the *caapi* plant cultivated among Amazonian peoples has ancestry linked to primordial humans. In Amazonian ontologies, people and objects share the same symbolic framework, existing as both things and embodied social relations. Powerful objects can engage in dialogues with humans, underscoring the significance of objects in Amerindian cosmologies, in which they are considered foundational elements of the world and its life forms.^100^

These understandings of the vegetal world resonate with broader critiques articulated by other Tukano intellectuals, most notably João Paulo Lima Barreto. A philosopher and anthropologist, Barreto pushes the reflection beyond the ontological status of individual plants or artifacts to interrogate the institutional frameworks in which these entities are preserved and displayed. Barreto considers that Western museums are not sites of conservation or curation, but instead *palácios dos mortos* – palaces of the dead – where living epistemic and cosmological relations are arrested and objectified.^101^

In such spaces, artifacts from the Upper Rio Negro region are not simply misplaced cultural items but severed cosmopolitical beings, cut off from the relational networks that gave them meaning, power, and ethical orientation. Once removed from their protocols of care, oral transmission, and cosmological activation, these objects do not merely lose function; they become inert, and potentially harmful. As Barreto poignantly observes, “even if we were to bring them back, they would no longer serve us.”^102^ This dislocation does not entail cultural nostalgia but ontological damage – an interruption of vitality that may result in imbalance, illness, or spiritual dissonance. What remains in the museum, then, are not heritage artifacts but cosmological corpses: fragments of disrupted worlds, stripped of their breath, detached from their ancestral moorings.^103^

Barreto’s critique moves beyond a politics of restitution and toward a more fundamental challenge to Western criteria for determining what qualifies as an epistemic object. His intervention is not to discard these collections but to reinscribe them within Indigenous systems of meaning, challenging dominant museological and scientific frameworks by foregrounding knowledge as activated through relation, narrative, and ceremony. Drawing on his dual experience as both an anthropologist and the son of shamanic leaders, Barreto advocates for a rethinking of the museum not as a neutral repository of knowledge, but as an active site of epistemicide – a concept he uses to describe the systematic dismantling of Indigenous knowledge systems through classificatory regimes alien to their origin.^104^ The taxonomies imposed on these objects, he argues, disarticulate living systems of thought and reduce dynamic cosmologies to aestheticized fragments or scientific specimens.^105^

Some of these insights emerged from recent conversations between Barreto and me in Manaus, one of the most important historical hubs of colonial and commercial activity in the Amazon. Once a key logistical center for naturalists such as Spruce, Wallace, and Martius during their nineteenth-century expeditions, Manaus remains today a vibrant intellectual crossroads. It is also the city where Barreto currently lives and teaches Indigenous anthropology. Our conversations, situated in a geography marked by historical extraction and ongoing resistance, touched on the shared experience of accessing archives and collections in the Global North – whether as Indigenous researchers or as Amazonian subjects navigating institutions shaped by colonial legacies. These exchanges lent further depth and resonance to his reflections on the epistemic afterlives of Amazonian artifacts, underscoring the asymmetries still embedded in the transit and interpretation of these materials.

Barreto’s argument culminates in the proposal of what might be called a contra-archive: not a countercollection in the conventional sense, but a radically different mode of engaging with material culture – one grounded in the reactivation of relational knowledge and cosmological accountability. In this framework, artifacts are not inert remnants of a past to be documented but vital agents capable of being reawakened through ceremonial and narrative protocols.^106^ This compels historians of science to interrogate not only the material contents of natural history and ethnographic museums, but also the epistemic infrastructures and colonial ontologies that have historically governed their practices of collection, classification, and display – regimes that continue to shape what counts as knowledge, who is authorized to produce it, and how objects are rendered intelligible or excluded within institutional narratives.^107^

Ultimately, Barreto’s intervention offers a powerful methodological and political proposition. If plants, artifacts, and other entities are to be taken seriously within their original ontological frameworks, then museum spaces must be reconceptualized as sites of ongoing epistemic struggle – not as final destinations for knowledge, but as unstable arenas in which multiple worlds collide, contend, and potentially negotiate reparative futures. His thought demands that we consider not only what is in the museum, but what is missing, silenced, or rendered unintelligible by its current forms. In this light, the museum becomes both a site of inquiry and a subject of critique – one that must be opened to Indigenous conceptual frameworks if it is to participate in the ethical work of epistemic redress.

## Final remarks

In this article, I have examined select objects housed in natural history and ethnographic museums in England and Portugal. Among these are wooden masks and plant fragments, artifacts imbued with layered meanings that transcend their immediate materiality. I have argued that these objects can be analyzed through two complementary lenses. Firstly, they can be historicized to uncover the forms of knowledge, power networks, and infrastructures that facilitated their extraction, abduction, or collection during scientific expeditions – a process of extensive and ongoing historical research.^108^ Secondly, the epistemological frameworks underpinning the creation of these artifacts can be explored through the conceptual lens of Amerindian perspectivism and the agency of objects, as previously discussed.

This second point is of fundamental importance – not only for conceptual clarity but also for historiographical reasons. While the study of material culture housed in archives and museums of the Global North has grown considerably, most works still fail to engage seriously with the epistemologies that originally shaped the creation and significance of these materials. This gap stems from multiple factors, including linguistic limitations, restricted access to Indigenous sources, and the enduring disciplinary boundaries between the history of science and anthropology.

This article addresses that omission by incorporating the perspectives of Indigenous intellectuals such as Kopenawa, Diakara, and João Paulo – not as cultural informants or brokers, but as theorists in their own right. While their reflections converge in critiquing colonial extraction and museological violence, they diverge in emphasis. Barreto offers a more radical stance: even restitution, he suggests, cannot fully repair broken relations. Kopenawa, by contrast, envisions the museum as a site of potential negotiation and epistemic repair. Diakara foregrounds the need for Indigenous curatorial agency and the reactivation of objects’ relational power.

Rather than collapsing these views into a unified model, I treat their divergences as part of a living, plural theorization of how material traces from the past might be resignified in the present. Their conceptual contributions are not ancillary; they challenge dominant epistemic regimes and provide historically grounded frameworks for understanding these objects. In doing so, the article advances a historiographical proposition: that the analysis of colonial collections must seriously engage the ontologies and cosmologies that once animated – and often continue to animate – their meanings.

This approach pushes beyond a critique of colonial extraction; it reveals how these specific objects index distinct but interwoven regimes of knowledge rooted in Indigenous cosmologies. The mask, in particular, is not merely decorative or illustrative; it embodies the capacity for transformation between human and animal worlds, as seen in shamanic visions where *xapiri* spirits take animal form.^109^ Placed behind glass, such an artifact is cut off from the relational and ceremonial context that animates it – its voice stilled, its shine extinguished. The plant, in turn, condenses a pharmacological and cosmopolitical history shaped by both Indigenous shamanic practices and modern bioprospecting. These materials demonstrate how museum collections preserve not only physical traces of colonial expeditions, but also unresolved tensions between radically different epistemic systems. These tensions demonstrate how the very notion of what constitutes an object – what is collectible, displayable, and knowable – remains entangled with ontological conflicts that these items still carry.

Such an approach not only brings alternative epistemologies to the forefront but also destabilizes established notions of boundaries between the human and nonhuman worlds. It challenges conventional understandings of space and time, shaped within the confines of Western paradigms and reinforced by modern scientific frameworks. In doing so, it invites historians to reassess their own methodological coordinates and analytical vocabularies. As argued in the introduction, this reorientation of the field – anchored in the concept of cosmo–historical juxtaposition – is not merely additive but transformative: it redefines the terms by which science, evidence, and knowledge could be historicized.

From a methodological perspective, it is essential to engage with scholars, artists, Indigenous and non-Indigenous voices who have underscored the explanatory power and richness of non-Western epistemologies. As historians of science and scholars of STS, we are well-situated to connect the fields of science, colonialism, and global history, facilitating a multifaceted understanding of the historical objects of “others” housed in the Global North. Nonetheless, a significant gap remains in incorporating Indigenous cosmologies into our analytical frameworks. The next step is to begin more comprehensively engaging with the Indigenous cosmologies and knowledge worlds that lent these materials interest and meaning in the first place.

This perspective is particularly relevant in the Amazon, one of the earliest regions colonized by Europeans, where Indigenous populations were long positioned at the intersection of modern scientific inquiry and theological judgment. However, they have increasingly asserted themselves as active participants in both Western and non-Western research processes and knowledge production. This discourse engages with the perspectives of the Yanomami shaman and the cosmological significance of objects from the Rio Negro, challenging how such artifacts are interpreted in Western institutions – including, but not limited to, collections at Kew Gardens and the Coimbra Science Museum.

The contributions of Davi Kopenawa and Jaime Diakara are central to this argument. Their reflections offer not only ethical or political interventions, but conceptual tools that challenge prevailing scientific and museological categories. Kopenawa’s notion of *xapiri* spirits – activated through ritual and deeply entangled with specific plants – reveals the inadequacy of Western dichotomies between nature and culture, or science and belief. Likewise, João Paulo’s work on materiality, ritual, and memory helps frame the museum not as a neutral repository, but as a site of persisting epistemic violence. Rather than treating these insights as merely illustrative, I suggest they offer generative frameworks through which to reinterpret historical objects. Their perspectives demonstrate that the presence of these objects in European institutions is not a closed chapter of scientific history, but an open epistemological dispute in which Indigenous theories of matter, spirit, and knowledge continue to contest colonial taxonomies.

Museums have long been regarded as pivotal arenas in the history of science, spaces where identities are shaped, memories are curated, and notions of truth about the external world are constructed and perpetuated.^110^ Museums operate on an epistemological plane, rendering visible the frameworks through which knowledge is organized and power enacted. Within this realm, the recognition of Indigenous epistemologies embedded in museum collections carries profound transformative potential. It offers a means to unsettle entrenched Western taxonomies, reimagine systems of categorization, and unravel the hierarchical architectures born of science’s historical pursuit of dominance and exploitation. In so doing, it opens up pathways for a more pluralistic and equitable understanding of the world, one that honors the multiplicity of voices and cosmologies silenced by the very institutions now called to bear witness to their resurgence.^111^

The distinction between “mere consultation” and “engagement with Indigenous epistemes” is essential here. Consultation often takes the form of simply involving Indigenous communities to validate or authenticate collections, without questioning the Western classificatory systems in which they have become entrenched. This approach, often labeled as “neo-colonial,” can perpetuate the very structures it seeks to rectify by treating Indigenous knowledge as an add-on rather than a foundational alternative to the Western scientific framework.^112^ Engagement with past and contemporary Indigenous epistemes involves a more profound, reciprocal exchange – one in which Indigenous ways of knowing and relating to the world are taken as legitimate, living systems of knowledge. This engagement means recognizing that Indigenous cosmologies and relationships with the material world – whether plants, animals, or artifacts – are dynamic and ongoing, resisting static classification or being subsumed under Western taxonomies.

This, in turn, raises another challenge: the potential conflict between historical objects and contemporary Indigenous conceptualizations and practices. There is a valid concern about presentism – assuming that Indigenous knowledge has remained unchanged over centuries. While it is crucial to avoid essentializing Indigenous knowledge as static, this does not mean that contemporary epistemologies cannot offer meaningful interpretations of historical objects. When it comes to contemporary epistemologies, it seems we already engage in such practices regularly – but predominantly with Western authors. We seldom encounter strong criticism of the relevance or suitability of employing thinkers like Benjamin, Habermas, Foucault, or Bourdieu to analyze the past, even when considering distant historical contexts.^113^ The same holds true when Western anthropologists produce knowledge about historical objects, often grounding their approaches in the aforementioned theorists.^114^ While it is valid to raise concerns about presentism and anachronism from a theoretical perspective, I argue that contemporary Indigenous epistemologies, particularly regarding the meanings and roles of historical objects, offer valuable and innovative angles for historical research.

Nonetheless, this invitation to take seriously the conceptual interventions of Indigenous intellectuals also requires a more critical reflection on the grounds of their authority. Figures such as Davi Kopenawa, Jaime Diakara, and João Paulo Barreto do not speak from a position of cultural transparency or essential identity. Their authority, while recognized within their own communities, is not naturalized in this analysis; rather, it emerges through complex articulations: between shamanic roles and ceremonial expertise, political activism and intellectual labor, and Indigenous forms of transmission and academic institutions.^115^ For example, Kopenawa’s epistemic position is shaped not only by his cosmological knowledge as a shaman but also by his coauthorship of *The Falling Sky* with the ethnologist Bruce Albert, which strategically mobilizes anthropological discourse to make his thought intelligible within a global arena. Barreto, in turn, inhabits a dual formation as shaman’s son and philosopher-anthropologist, enabling him to formulate critiques of museums and archives that are at once rooted in Tukano cosmology and engaged with Western epistemological debates.

This position of “speaking for” is not without tensions. As Daniel Fabre^116^ has suggested through the notion of hommes-monde, such intellectuals often become authorized to represent not only themselves but an entire people or cosmology. While this role may offer a powerful counterpoint to colonial narratives, it also risks reactivating problematic assumptions – such as the idea that Indigenous peoples speak from a timeless cultural core or that their knowledge remains unchanged across centuries.^117^ It is precisely to avoid such essentializations that their interventions must be read not as fixed representations of culture, but as historically situated, politically charged, and epistemologically generative.

The strength of these perspectives lies not in recovering an originary meaning, but in their ability to reframe our relation to the past through cosmopolitical critique. What they offer is not a reified image of “Indigenous culture,” but a methodological proposition: that knowledge resides not only in archives or vitrines, but in relations, narratives, and obligations that endure through other temporalities.

In this light, the interventions of contemporary Indigenous thinkers are not merely commentaries on historical artifacts; they are active contributions to ongoing disputes over meaning, memory, and materiality – what we might call epistemological acts of insurgency. At the same time, historical analysis can offer something back to these theoretical frameworks: attention to temporal layers, archival sedimentation, and the ways colonial epistemologies operated not as abstract systems but as situated practices. This historiographical lens may help refine the critical tools Indigenous thinkers already mobilize with force and clarity.

By acknowledging both the enduring continuities and shifting transformations in the production of scientific knowledge, public institutions – including, but not limited to, museums – can help foster intellectual and cultural landscapes that engage meaningfully with the ongoing struggles of Indigenous communities. This includes affirming their cultural sovereignty while also attending to the profound historical resonance of the objects under their care. As Tuck and Yang remind us, decolonization is not a metaphor – it entails concrete struggles for land and political representation.^118^ These dimensions must be intrinsically connected to the histories we tell in museums, films, and scientific writing.

The critical question, then, is to what extent historians of science acknowledge these stakes – and whether we are prepared, or genuinely committed, to advance such an agenda. This is not a blueprint to be applied across contexts. Rather, this article offers a methodological and historiographical proposition: an invitation to take Indigenous conceptual labor seriously. It proposes a critical and open-ended approach – not a definitive framework – with the aim of opening up routes for future work where historical, curatorial, and Indigenous perspectives may converge in more plural, collaborative, and cosmopolitical ways.

## Footnotes

1. Davi Kopenawa and Bruce Albert, *A Queda do Céu: Palavras de um Xamã Yanomami* (São Paulo: Companhia das Letras, 2010), p.345.

2. Walter Mignolo, “Museums in the Colonial Horizon of Modernity,” in Jonathan Harris (ed.) *Globalization and Contemporary Art* (Hoboken, NJ: Wiley Blackwell, 2011), pp.71–86; Joanna Page, *Decolonizing Science in Latin American Art* (London: UCL Press, 2021). Laura Peers and Alison Brown (eds.), *Museums and Source Communities: A Routledge Reader* (London: Routledge, 2003); Christina Kreps, *Liberating Culture: Cross-Cultural Perspectives on Museums, Curation, and Heritage Preservation* (London: Routledge, 2003); Linda T. Smith, *Decolonizing Methodologies: Research and Indigenous Peoples* (London: Zed Books, 1999).

3. Eduardo Viveiros de Castro, *A Inconstância da Alma Selvagem* (São Paulo: Cosac Naify, 2002); Eduardo Viveiros de Castro, *Metafísicas canibais: Elementos para uma antropologia pós-estrutural* (São Paulo: Ubu Editora, 2018); Philippe Descola, *Beyond Nature and Culture* (Chicago, IL: University of Chicago Press, 2013).

4. Lynette Russell, “Indigenous Knowledge and Archives: Accessing Hidden History and Understandings,” *Australian Academic & Research Libraries* 36, no. 2 (2005): 161–71; Ann Laura Stoler, *Along the Archival Grain: Epistemic Anxieties and Colonial Common Sense* (Princeton, NJ: Princeton University Press, 2010).

5. This observation is primarily based on studies focused on Amazonian cases. One exception is Neil Safier, “Masked Observers and Mask Collectors: Entangled Visions from the Eighteenth-Century Amazon,” *Colonial Latin American Review* 26, no. 1 (2017): 104–30; For histories of science beyond the Amazon that analyze Indigenous epistemologies and material culture, see, for instance, Efram Sera-Shriar, “From Museumization to Decolonization: Fostering Critical Dialogues in the History of Science with a Haida Eagle Mask,” *British Journal for the History of Science* 56, no. 3 (2023): 309–28. In contrast, the fields of anthropology, museology, and archaeology feature a significantly larger volume of work on these topics.

6. Fernando Santos-Granero, *The Occult Life of Things: Native Amazonian Theories of Materiality and Personhood* (Tucson, AZ: University of Arizona Press, 2009).

7. Geoffrey Lloyd’s works marks significant milestones in the development of this field. See, for example, G. E. R. Lloyd, *Expanding Horizons in the History of Science* (Cambridge: Cambridge University Press, 2021); G. E. R. Lloyd, *The Ambivalences of Rationality: Ancient and Modern Cross-Cultural Explorations* (Cambridge: Cambridge University Press, 2017).

8. This question – of how imperial knowledge regimes engaged with, transformed, or suppressed non-European epistemologies – has been widely discussed in the historiography. See, for example, Simon Schaffer et al. (eds.), *The Brokered World: Go-Betweens and Global Intelligence, 1770–1820* (Sagamore Beach, MA: Science History Publications, 2009); Kapil Raj, *Relocating Modern Science: Circulation and the Construction of Knowledge in South Asia and Europe, 1650–1900* (New York, NY: Palgrave Macmillan, 2007); Londa Schiebinger, *Plants and Empire: Colonial Bioprospecting in the Atlantic World* (Cambridge, MA: Harvard University Press, 2004).

9. Sandra Harding, *Objectivity and Diversity: Another Logic of Scientific Research* (Chicago, IL: University of Chicago Press, 2011); Eduardo Kohn, *How Forests Think: Toward an Anthropology Beyond the Human* (Berkeley, CA: University of California Press, 2013); Amiria Henare, Martin Holbraad, and Sari Wastell (eds.), *Thinking Through Things: Theorising Artefacts Ethnographically* (London: Routledge, 2007).

10. Santos-Granero, *The Occult Life of Things* (note 6); Henare et al., *Thinking Through Things* (note 9).

11. Nicholas Thomas, *Entangled Objects: Exchange, Material Culture, and Colonialism in the Pacific* (Cambridge, MA: Harvard University Press, 1991); Various Authors, “Entangled Histories, Entangled Worlds: Reflections on Time, Space, and Place,” *Global History Quarterly* 5, no. 3 (2018): 45–67; Gianpaolo Chiriacò, Gerhild Fuchs, and Birgit Mertz-Baumgartner, “Entangled Histories and Voices: An Introduction,” *ATeM Journal* 2, no. 1 (2022): 1–5.

12. Prominent venues in which these Indigenous artists have showcased their work and critiques include the Venice Biennale and the São Paulo Biennial. I refer specifically to the works of artists Denilson Baniwa and Uýra Sodoma. The former delves into colonial archives, creating interventions and reinterpretations of canonical works and images from Brazil’s colonial history, such as those by Padre António Vieira, de Bry, and Rugendas. Uýra Sodoma, a biologist by training, incorporates themes of historical ecology into her interventions in Amazonian cities such as Manaus and Belém.

13. Moira G. Simpson, *Making Representations: Museums in the Post-Colonial Era* (London: Routledge, 2007).

14. Davi Kopenawa and Bruce Albert, *The Falling Sky: Words of a Yanomami Shaman* (Cambridge, MA: Belknap Press, 2015).

15. Ramón Grosfoguel, “Decolonizing Post Colonial Studies and Paradigms of Political Economy: Transmodernity, Decolonial Thinking, and Global Coloniality,” *TransModernity* 1, no. 1 (2011): 40–68; Aníbal Quijano, “Coloniality of Power, Eurocentrism, and Latin America,” *Nepantla: Views from South* 1, no. 3 (2000): 533–80; Walter D. Mignolo and Catherine Walsh, *On Decoloniality: Concepts, Analytics, Praxis* (Durham, NC: Duke University Press, 2018).

16. Viveiros de Castro, *A Inconstância da Alma Selvagem* (note 3).

17. Santos-Granero, *The Occult Life of Things* (note 6).

18. Viveiros de Castro, *Metafísicas canibais* (note 3).

19. Descola, *Beyond Nature and Culture* (note 3).

20. This critique aligns with Latin American decolonial epistemologies – emphasized by Sandra Harding’s analysis of feminist and decolonial philosophies of science – and foundational decolonial concepts such as *coloniality of power* (Quijano), *epistemic disobedience* (Mignolo), *pluriversal thinking* (Mignolo & Walsh), and *ontological pluralism* (Escobar). It also resonates with recent comparative metaphysical approaches that seek to move beyond Western ontologies and recognize multiple modes of world-making. See Sandra Harding, “State of the Field: Latin American Decolonial Philosophies of Science,” *Studies in History and Philosophy of Science Part A* 78 (2019): 48–63; Quijano, “Coloniality of Power,” (note 15); Mignolo and Walsh, *On Decoloniality* (note 15); Arturo Escobar, *Designs for the Pluriverse: Radical Interdependence, Autonomy, and the Making of Worlds* (Durham, NC: Duke University Press, 2018); Pierre Charbonnier, Gildas Salmon, and Peter Skafish (eds.), *Comparative Metaphysics: Ontology After Anthropology*, Kindle edition (Lanham, MD: Rowman & Littlefield, 2016).

21. Safier, “Masked Observers and Mask Collectors,” (note 5); Tabitha Cadbury, “Imagining the Amazon: The Whiffen Collection at Cambridge,” *Journal of Museum Ethnography*, no. 16 (2004): 85–100; Christian Feest, “Maximilian Prince of Wied-Neuwied and His Ethnographic Collection from Eastern Brazil, 1815–1817,” *Indiana* 37, no. 2 (2020): 47–69; Mariana Françozo and Maria Patricia Ordoñez, “Introduction to the Special Issue ‘Collecting Latin America in the Nineteenth Century’,” *Museum History Journal* 12 (2019): 1–6.

22. John Hemming, *Tree of Rivers: The Story of the Amazon* (London: Thames & Hudson, 2008); David Cleary, “Towards an Environmental History of the Amazon: From Prehistory to the Nineteenth Century,” *Latin American Research Review* 36, no. 2 (2001): 64–96.

23. Marianne Schmink and Charles H. Wood, *Contested Frontiers in Amazonia* (New York, NY: Columbia University Press, 1992).

24. Irene Fattacciu, *Empire, Political Economy, and the Diffusion of Chocolate in the Atlantic World* (New York, NY: Routledge, 2020).

25. Jessica Hernández, *Fresh Banana Leaves: Healing Indigenous Landscapes Through Indigenous Science* (Seattle, WA: University of Washington Press, 2022); Camilla Townsend, *Fifth Sun: A New History of the Aztecs* (Oxford: Oxford University Press, 2019).

26. Stuart B. Schwartz, *Slaves, Peasants, and Rebels: Reconsidering Brazilian Slavery* (Urbana, IL: University of Illinois Press, 1992).

27. Warren Dean, *With Broadax and Firebrand: The Destruction of the Brazilian Atlantic Forest* (Berkeley, CA: University of California Press, 1995).

28. Rafael Chambouleyron, *As Drogas do Sertão e a Amazônia Colonial Portuguesa* (Lisboa: Centro de História da Universidade de Lisboa, 2023).

29. Hemming, *Tree of Rivers* (note 22); Dauril Alden, *The Making of an Enterprise: The Society of Jesus in Portugal, Its Empire, and Beyond, 1540–1750* (Stanford, CA: Stanford University Press, 1996).

30. John M. Monteiro, *Negros da Terra: Índios e Bandeirantes nas Origens de São Paulo* (São Paulo: Companhia das Letras, 2001).

31. Heather Roller, *Amazonian Routes: Indigenous Mobility and Colonial Communities in Northern Brazil* (Stanford, CA: Stanford University Press, 2014).

32. Safier, “Masked Observers and Mask Collectors,” (note 5); Schiebinger, *Plants and Empire* (note 8).

33. João Daniel, *Tesouro Descoberto no Máximo Rio Amazonas*, 2 vols. (Rio de Janeiro: Contraponto, 2004).

34. Luciana Osorio Sunnucks et al., “Amazonian Activism for European Museum Audiences: Critical Reflections on the Development of an ‘Amazonia’ Exhibition at the National Museum of World Cultures, the Netherlands,” *Curator* 66, no. 2 (2023): 277–95; Hugh Raffles, *In Amazonia: A Natural History* (Princeton, NJ: Princeton University Press, 2002).

35. Raphael Uchôa, “‘Savage Knowledge,’ Ethnosciences, and the Colonial Ways of Producing Reservoirs of Indigenous Epistemologies in the Amazon,” *Journal of Social Ontology* 10, no. 2 (2024): 1–23.

36. Feest, “Maximilian Prince of Wied-Neuwied,” (note 21).

37. Tony Bennett, *The Birth of the Museum: History, Theory, Politics* (London: Routledge, 1995); Johannes Fabian, *Time and the Other: How Anthropology Makes Its Object* (New York, NY: Columbia University Press, 1983).

38. Daniela Bleichmar, *Visible Empire: Botanical Expeditions and Visual Culture in the Hispanic Enlightenment* (Chicago, IL: University of Chicago Press, 2012); Sam Alberti, “Objects and the Museum,” *Isis* 96, no. 4 (2005): 559–71.

39. Amy Lonetree, *Decolonizing Museums: Representing Native America in National and Tribal Museums* (Chapel Hill, NC: University of North Carolina Press, 2012).

40. M. L. Rodrigues de Areia, Maria Arminda Miranda, and Tekla Hartmann (eds.), *Memória da Amazónia: Alexandre Rodrigues Ferreira e a Viagem Philosophica pelas Capitanias do Grão-Pará, Rio Negro, Mato Grosso e Cuyabá, 1783–1792* (Coimbra: Museu e Laboratório Antropológico, Universidade de Coimbra, 1991).

41. Ibid.

42. João Carlos Pires Brigola, *Colecções, Gabinetes e Museus em Portugal no Século XVIII* (Lisboa: Fundação Calouste Gulbenkian, 2003); Carlos Fiolhais, Carlota Simões e Décio Martins (eds.), *História da Ciência na Universidade de Coimbra, 1772–1933* (Coimbra: Imprensa da Universidade de Coimbra, 2013).

43. Márcia H. M. Ferraz, *As Ciências em Portugal e no Brasil (1772–1822): O Texto Conflituoso da Química* (São Paulo: Educ, Fapesp, 1997).

44. Kenneth Maxwell, *Pombal: Paradox of the Enlightenment* (Cambridge: Cambridge University Press, 2003); Francisco Bethencourt and José Curto (eds.), *Portuguese Oceanic Expansion, 1400–1800* (Cambridge: Cambridge University Press, 2007); Carlos Fiolhais, Carlota Simões, and Décio Martins (eds.), *História da Ciência na Universidade de Coimbra, 1772–1933* (Coimbra: Imprensa da Universidade de Coimbra, 2013).

45. Museu da Ciência da Universidade de Coimbra, “Gabinete de Curiosidades – Uma Interpretação,” 12 August 2024, https://www.museudaciencia.org/index.php?module=events&option=calendar&id=1114 (accessed 23 August 2025); João Brigola e Paulo Trincão, “O Gabinete de Curiosidades da Universidade de Coimbra: uma proposta museológica e científica,” *História, Ciências, Saúde – Manguinhos* 30 (2023): 1–14.

46. Paula Findlen, *Possessing Nature: Museums, Collecting, and Scientific Culture in Early Modern Italy* (Berkeley, CA: University of California Press, 1994).

47. Sharon Macdonald, *Memorylands: Heritage and Identity in Europe Today* (London: Routledge, 2003); James Clifford, *The Predicament of Culture: Twentieth Century Ethnography, Literature, and Art* (Cambridge, MA: Harvard University Press, 1988); Tony Bennett, *Pasts Beyond Memory: Evolution, Museums, Colonialism* (London: Routledge, 2004); Sharon Macdonald (ed.), *The Politics of Display: Museums, Science, Culture* (London: Routledge, 2003).

48. Rodrigues de Areia et al., *Memória da Amazónia* (note 40).

49. Kopenawa and Albert, *A Queda do Céu* (note 1).

50. Ibid.

51. Ibid., pp.194–5.

52. Ibid.

53. Ibid., p.72.

54. Ibid., p.146.

55. Marisol de la Cadena, *Earth Beings: Ecologies of Practice across Andean Worlds* (Durham, NC: Duke University Press, 2015); Eduardo Viveiros de Castro, “Cosmological Perspectivism in Amazonia and Elsewhere,” *HAU Masterclass Series* 1 (1998): 1–22.

56. Bruce Albert and Davi Kopenawa, *O espírito da floresta* (São Paulo: Companhia das Letras, 2023).

57. Kopenawa and Albert, *A Queda do Céu*, p.816 (note 1).

58. Albert and Kopenawa, *O espírito da floresta* (note 56).

59. Kopenawa and Albert, *A Queda do Céu*, pp.587–8 (note 1).

60. Benjamin Alberti and Tamara L. Bray (eds.), *Animating Archaeology: Of Subjects, Objects and Alternative Ontologies* (Oxford: Oxbow Books, 2009).

61. Kopenawa and Albert, *A Queda do Céu*, pp.587–90 (note 1).

62. Shira Shmuely, “Curare: The Poisoned Arrow That Entered the Laboratory and Sparked a Moral Debate,” *Social History of Medicine* 33, no. 3 (2020): 881–97; Bruno Latour, “Circulating Reference: Sampling the Soil in the Amazon Forest,” in *Pandora’s Hope: Essays on the Reality of Science Studies* (Cambridge, MA: Harvard University Press, 1999), pp.24–79. Bruno Latour, *Pandora’s Hope: Essays on the Reality of Science Studies* (Cambridge, MA: Harvard University Press, 1999), pp.24–79.

63. Karen Shiratori, “Tempo e evento na onirocrítica ameríndia: um balanço bibliográfico,” *Revista de Antropologia* 65, no. 3 (2022): e195928; Karen Shiratori et al., “Novas perspectivas sobre os sonhos ameríndios: uma apresentação,” *Revista de Antropologia* 65 (2022): e202767.

64. Harding, *Objectivity and Diversity* (note 9).

65. Kopenawa and Albert, *A Queda do Céu*, pp.587–90 (note 1).

66. Ibid.

67. Ibid.

68. Ibid.

69. Ibid.

70. Lorraine Daston and Peter Galison, *Objectivity* (New York, NY: Zone Books, 2007); Donna Haraway, “Situated Knowledges: The Science Question in Feminism and the Privilege of Partial Perspective,” *Feminist Studies* 14, no. 3 (1988): 575–99.

71. Kopenawa and Albert, *A Queda do Céu*, p.109 (note 1).

72. Ibid., p.447.

73. Ibid., p.455.

74. Steven Shapin, *A Social History of Truth: Civility and Science in Seventeenth-Century England* (Chicago: University of Chicago Press, 1994), pp.355–408. Haraway, “Situated Knowledges,” 575–99 (note 70).

75. Kopenawa and Albert, *A Queda do Céu*, pp.587–90 (note 1).

76. Ibid.

77. Samuel J. Redman, *Prophets and Ghosts: The Story of Salvage Anthropology*, Kindle edition (Cambridge, MA: Harvard University Press, 2021); Thomas Biolsi and Larry J. Zimmerman (eds.), *Indians and Anthropologists: Vine Deloria Jr. and the Critique of Anthropology* (Tucson, AZ: University of Arizona Press, 1997); Raphael Uchôa, “From the State of Nature to the State of Ruins: ‘American Race’ and ‘Savage Knowledge’ According to Carl von Martius,” *Annals of Science* 79, no. 1 (2022): 1–25.

78. Kopenawa and Albert, *A Queda do Céu*, p.592 (note 1).

79. Donna Haraway, “Situated Knowledges: The Science Question in Feminism and the Privilege of Partial Perspective,” *Feminist Studies* 14, no. 3 (1988): 575–99; Stoler, *Along the Archival Grain* (note 4); Michel-Rolph Trouillot, *Silencing the Past: Power and the Production of History* (Boston, MA: Beacon Press, 1995); Sandra Harding, *Whose Science? Whose Knowledge? Thinking from Women’s Lives* (Ithaca, NY: Cornell University Press, 1991); Linda Tuhiwai Smith, *Decolonizing Methodologies: Research and Indigenous Peoples*, 2nd ed. (London: Zed Books, 2012).

80. John Hemming, *Naturalists in Paradise: Wallace, Bates and Spruce in the Amazon* (New York, NY: Thames & Hudson, 2015); Peter Raby, *Bright Paradise: Victorian Scientific Travellers* (Princeton, NJ: Princeton University Press, 2001).

81. Leslie Bethell, *Brazil: Empire and Republic, 1822–1930* (Cambridge: Cambridge University Press, 1989).

82. Leslie Bethell, *Brazil: Essays on History and Politics* (London: University of London Press, 2018); Steven Topik, Carlos Marichal, and Zephyr Frank (eds.), *From Silver to Cocaine: Latin American Commodity Chains and the Building of the World Economy, 1500–2000* (Durham, NC: Duke University Press, 2006).

83. Barbara Weinstein, *The Amazon Rubber Boom, 1850–1920* (Stanford, CA: Stanford University Press, 1983).

84. Uchôa, “‘Savage Knowledge,’ Ethnosciences” (note 35).

85. Richard Drayton, *Nature’s Government: Science, Imperial Britain, and the ‘Improvement’ of the World* (New Haven, CT: Yale University Press, 2000); Felix Driver, Mark Nesbitt, and Caroline Cornish (eds.), *Mobile Museums: Collections in Circulation* (London: UCL Press, 2021); Lucile H. Brockway, *Science and Colonial Expansion: The Role of the British Royal Botanic Gardens* (New Haven, CT: Yale University Press, 2002).

86. Uchôa, “‘Savage Knowledge,’ Ethnosciences” (note 35).

87. Richard Spruce to Sir William Jackson Hooker, June 27, 1853, Library and Archives at Royal Botanic Gardens, Kew, Directors’ Correspondence 71/368.

88. Richard Spruce to George Bentham, November 2, 1858, in A. R. Wallace (ed.), *Notes of a Botanist on the Amazon and Andes*, 2 vols. (London: Macmillan, 1908), pp.217–8.

89. Ibid.

90. Robin M. Wright (ed.), *História Indígena e do Indigenismo no Alto Rio Negro* (Campinas, SP: Editora Mercado de Letras, 2005).

91. Hemming, *Naturalists in Paradise* (note 80).

92. Stephen Hugh-Jones, *The Palm and the Pleiades: Initiation and Cosmology in Northwest Amazonia* (Cambridge: Cambridge University Press, 1979); Santos-Granero, *The Occult Life of Things* (note 6).

93. Jaime Diakara, *Gaapi: Uma viagem por este e outros mundos* (Manaus: Valer Editora, 2023), pp.42–3.

94. Ibid.

95. Ibid., 44.

96. Eduardo Viveiros de Castro, *Metafísicas Canibais* (São Paulo: Cosac Naify, 2009).

97. Pierre Charbonnier, Gildas Salmon, and Peter Skafish (eds.), *Comparative Metaphysics: Ontology After Anthropology* (London: Rowman & Littlefield Publishers, 2017); César E. Giraldo Herrera, *Microbes and Other Shamanic Beings* (Cham, Switzerland: Palgrave Macmillan, 2018).

98. Santos-Granero, *The Occult Life of Things* (note 6).

99. Arjun Appadurai (ed.), *The Social Life of Things: Commodities in Cultural Perspective* (Cambridge: Cambridge University Press, 1986).

100. Santos-Granero, *The Occult Life of Things* (note 6).

101. João Paulo Lima Barreto, *Palácio dos Mortos* [Palace of the Dead] (unpublished manuscript, Manaus: Universidade Federal do Amazonas, 2014).

102. Ibid., p.12.

103. João Paulo Lima Barreto and A. Ekman, “A Amazônia nos porões dos ‘palácios dos mortos’,” *Humboldt – Magazin*, Goethe-Institut, June 2025.

104. Ibid.

105. Ibid.

106. Barreto, *Palácio dos Mortos* (note 101).

107. Ibid.

108. Roy MacLeod (ed.), *Osiris*, vol. 15, *Nature and Empire: Science and the Colonial Enterprise* (Chicago, IL: University of Chicago Press, 2000); Richard Drayton, *Nature’s Government: Science, Imperial Britain, and the ‘Improvement’ of the World* (New Haven, CT: Yale University Press, 2000); Banu Subramaniam, *Botany of Empire: Plant Worlds and the Scientific Legacies of Colonialism* (Durham, NC: Duke University Press, 2019; repr., 2023); Schiebinger, *Plants and Empire* (note 8).

109. Kopenawa and Albert, *A Queda do Céu*, pp.194–5 (note 1).

110. Bennett, *Pasts Beyond Memory* (note 47); Sharon Macdonald (ed.), *The Politics of Display: Museums, Science, Culture* (London: Routledge, 2003).

111. There are now numerous academic works and artistic exhibitions that provide insights into how Indigenous knowledge worlds and cosmologies could be integrated into the formation of scientific knowledge, museum exhibitions, and beyond. With regard to the Amazonian context see, for instance, Denilson Baniwa, *Muputyra. Nascer em flores* (Rio de Janeiro: Museu do Jardim Botânico, 2023); Daiara Tukano, *Nhe’ẽ Porã: Memória e Transformação* (Belém: Museu Paraense Emílio Goeldi, 2024); Lúcia Hussak van Velthem, K. Kukawka, e L. Joanny, “Museus, coleções etnográficas e a busca do diálogo intercultural,” *Boletim do Museu Paraense Emílio Goeldi. Série Ciências Humanas* 12 (2017): 101–14.

112. Lonetree, *Decolonizing Museums* (note 39); Alberti and Bray (eds.), *Animating Archaeology* (note 60).

113. Susan Buck-Morss, *The Dialectics of Seeing: Walter Benjamin and the Arcades Project* (Cambridge, MA: MIT Press, 1991). David Carrier, *A World Art History and Its Objects* (University Park, PA: Penn State University Press, 2008). Bennett, *The Birth of the Museum* (note 37). Sharon Macdonald (ed.), *A Companion to Museum Studies* (Malden, MA: Wiley-Blackwell, 2006).

114. Talal Asad (ed.), *Anthropology & the Colonial Encounter* (New York, NY: Humanity Books, 1973); Ivan Karp and Steven D. Lavine (eds.), *Exhibiting Cultures: The Poetics and Politics of Museum Display* (Washington, DC: Smithsonian Institution Press, 1991); Clifford, *The Predicament of Culture* (note 47).

115. Albert and Kopenawa, *O espírito da floresta* (note 56).

116. Daniel Fabre, “L’homme-monde.” *L’Homme* 1, no. 185–186 (2008): 309–22.

117. Clifford, *The Predicament of Culture* (note 47).

118. Eve Tuck and K. Wayne Yang, “Decolonization is Not a Metaphor,” *Decolonization: Indigeneity, Education & Society* 1, no. 1 (2012): 1–40.

